# Genes Contributing to Pain Sensitivity in the Normal Population: An Exome Sequencing Study

**DOI:** 10.1371/journal.pgen.1003095

**Published:** 2012-12-20

**Authors:** Frances M. K. Williams, Serena Scollen, Dandan Cao, Yasin Memari, Craig L. Hyde, Baohong Zhang, Benjamin Sidders, Daniel Ziemek, Yujian Shi, Juliette Harris, Ian Harrow, Brian Dougherty, Anders Malarstig, Robert McEwen, Joel C. Stephens, Ketan Patel, Cristina Menni, So-Youn Shin, Dylan Hodgkiss, Gabriela Surdulescu, Wen He, Xin Jin, Stephen B. McMahon, Nicole Soranzo, Sally John, Jun Wang, Tim D. Spector

**Affiliations:** 1 Department of Twin Research and Genetic Epidemiology, King's College London, London, United Kingdom; 2 Pfizer Limited, Neusentis, Granta Park, Cambridge, United Kingdom; 3 BGI–Shenzhen, Shenzhen, China; 4 Wellcome Trust Sanger Institute, Wellcome Trust Genome Campus, Hinxton, United Kingdom; 5 Pfizer Research Laboratories, Groton, Connecticut, United States of America; 6 Worldwide R&D, Pfizer, Cambridge, Massachusetts, United States of America; 7 Pfizer Global Research and Development, Sandwich, United Kingdom; 8 School of Bioscience and Biotechnology, South China University of Technology, Guangzhou, China; 9 Wolfson CARD, King's College London, London, United Kingdom; 10 Novo Nordisk Foundation Center for Basic Metabolic Research, University of Copenhagen, Copenhagen, Denmark; 11 Department of Biology, University of Copenhagen, Copenhagen, Denmark; Baylor College of Medicine, United States of America

## Abstract

Sensitivity to pain varies considerably between individuals and is known to be heritable. Increased sensitivity to experimental pain is a risk factor for developing chronic pain, a common and debilitating but poorly understood symptom. To understand mechanisms underlying pain sensitivity and to search for rare gene variants (MAF<5%) influencing pain sensitivity, we explored the genetic variation in individuals' responses to experimental pain. Quantitative sensory testing to heat pain was performed in 2,500 volunteers from TwinsUK (TUK): exome sequencing to a depth of 70× was carried out on DNA from singletons at the high and low ends of the heat pain sensitivity distribution in two separate subsamples. Thus in TUK1, 101 pain-sensitive and 102 pain-insensitive were examined, while in TUK2 there were 114 and 96 individuals respectively. A combination of methods was used to test the association between rare variants and pain sensitivity, and the function of the genes identified was explored using network analysis. Using causal reasoning analysis on the genes with different patterns of SNVs by pain sensitivity status, we observed a significant enrichment of variants in genes of the angiotensin pathway (Bonferroni corrected p = 3.8×10^−4^). This pathway is already implicated in animal models and human studies of pain, supporting the notion that it may provide fruitful new targets in pain management. The approach of sequencing extreme exome variation in normal individuals has provided important insights into gene networks mediating pain sensitivity in humans and will be applicable to other common complex traits.

## Author Summary

Chronic widespread pain is a complex clinical problem. Identification of underlying genetic factors would shed light on the biology of pain and offer targets for novel therapies. We aimed to identify rare genetic variants in the normal population associated with pain sensation by performing exome sequencing on individuals who were more or less sensitive to heat pain. While we did not identify any single variants having large effect, we did observe major group differences between the sensitive and insensitive individuals. Network analysis suggested a role for the angiotensin pathway, which previous work in animal models has suggested is important in pain mediation. Our results cast light on the genetic factors underlying normal pain sensation in humans and the utility of exome analyses. It suggests that further exploration of the angiotensin pathway may reveal novel targets for the treatment of pain.

## Introduction

Chronic pain has a prevalence of nearly 20% in Europe [1] and similar estimates are reported for North America. The symptom is poorly controlled by existing therapies and the resulting personal and socio-economic burden is considerable. While many analgesic drugs are available, the vast majority of analgesic prescriptions are drawn from two classes of drug, opiates and nonsteroidal anti-inflammatory-like drugs, and have either limited efficacy or significant side effects. There is, therefore, a considerable need to develop novel analgesic treatments. The use of human genetics for identification of intrinsic factors that contribute to chronic pain states is attractive for several reasons. Chronic pain conditions as well as experimentally induced pain have been shown to have a considerable genetic component [2]. Twin studies have shown observed heritabilities of about 50% for different pain traits [3]. The manifestation of pain in response to experimental stimuli such as skin heating, or to clinical pathologies such as joint degeneration, is known to vary markedly. It is clear that a range of factors, including personality, expectation and mental state modulate the expression of chronic pain and these features are themselves genetically mediated. Modelling in twins, however, suggests that there are two separate predisposing genetic factors [4] including variants that modulate sensitivity to pain, as well as those mediating anxiety and depression. A number of approaches to pain sensitivity genetics have been adopted including the examination of rare (monogenic) syndromes of pain insensitivity (reviewed in [5]) and candidate genes identified from transcriptional profiling in animal models [6]. Candidate gene studies in humans with chronic pain have been unconvincing, and confirmed candidate gene associations are still lacking (reviewed in [4] and [7]). The aim of the present study was to examine the influence of genetic variation, particularly rare variants having minor allele frequency <5%, on pain sensitivity in normal human volunteers. Two hypotheses were tested; that a single rare variant having large effect influences pain sensitivity and that the burden of variation would differ between sensitive and insensitive individuals.

Attempts to standardise and quantify pain sensibility in humans have led to the introduction of standardised thermal, mechanical or chemical stimuli that activate the nociceptive (pain signalling) system. Such quantitative sensory testing (QST) has been used to show that an individual's sensitivity to experimental pain predicts risk of developing chronic pain after surgical interventions such as hernia repair [8] and arthroscopy [9]. That pre-operative pain sensitivity is a major risk factor for chronic post-operative pain suggests that exploration of genetic variation underlying experimental pain might be a useful approach. The pain stimulus, its site of application and methods of rating have all been standardised - unlike spontaneous pain in a disease state. A further benefit is that the genetic influence on pain sensitivity is studied, rather than its influences on disease and disease progression. In the present study, we sought to determine whether rare variants associate with extremes of pain sensitivity in healthy volunteers. Using heat as the stimulus for QST in a large sample of healthy twin volunteers (www.twinsuk.ac.uk) we observed the normal variation in pain sensitivity using two objective tests, the heat pain threshold (HPT) and the heat pain suprathreshold (HPST). From a study population of >2500 individuals having QST, we compared approximately 200 individuals categorised as having high and low sensitivity to HPST (approximately 100 from each; TUK1 set) then repeated the process in a further 200 individuals (TUK2 set).

Our initial analysis sought to identify genes harbouring single nucleotide variants (SNVs) in either pain sensitive or insensitive subjects, with a focus on non-synonymous exonic and nonsense mutations. A large number of methods have been proposed for such an analysis [40]–[44]. We employed a battery of such tests including both old and new techniques, as well as tests examining a range of hypotheses; a difference between pain groups (sensitive vs. insensitive) in the proportion of subjects harbouring rare variants; a difference in abundance of rare variants, weighted by function; and a multivariate difference in variant patterns between the two groups, allowing simultaneous excess in either pain group for any single rare variant within a gene.

We found no single rare variant to have a statistically significant association to heat sensitivity, after multiple testing correction. The strongest signal was found for GZMM, a serine protease from immune cell granules. However, our network analysis identified up to 30 genes harbouring rare SNVs as belonging to the Angiotensin II pathway, which has previously been linked to the pain phenotype in a number of settings.

## Results

Singleton females were drawn from same sex twin pairs included in the sample so that gender- and relatedness bias were removed; after quality control all subjects were of north European descent. Complete data were available on 413 singleton subjects: TUK1 comprised 203 and TUK2 210 individuals. Analysis was performed in stages: TUK1, TUK2, combined TUK1 and TUK2 and pathway analysis. Details of the study participants are shown in Table 1. Based on the sample size required for exome sequencing and on the distributions obtained for HPT and HPST, insensitivity to heat pain was defined as HPST≥49.2°C, and sensitivity as HPST≤45.5°C. An individual designated insensitive/sensitive on HPST was included only if their HPT measure was higher/lower than the median HPT (46.6°C). The distributions of the TUK2 set were somewhat shifted, with median HPT = 46.0°C. Insensitivity to pain in TUK2 was defined as HPST> = 48.9°C while sensitivity was defined as HPST< = 45.4°C, with subjects required to have HPT above/below 46.0°C, respectively. Description of the exome sequencing findings in TUK1 and TUK2 groups is shown in Table S1. Details of the SNVs identified in the 2 datasets are shown in Table 2. The TUK2 set identified more variants of all types (except partial codons, which were extremely infrequent), which likely reflected the different exome capture arrays used and was consistent with greater coverage captured for the TUK2 set. However, the HapMap samples (n = 3) duplicated in the TUK1 and TUK2 exome sequencing showed no significant difference between number of SNVs called by the two platforms on commonly captured regions (by paired t-test, p = 0.24). The relative frequencies of the variants identified in the 2 datasets were compared to those recorded in dbSNP (http://www.ncbi.nlm.nih.gov/projects/SNP/) (Figure S1). Unsurprisingly, the majority of novel SNVs identified were rare, with estimated minor allele frequency (MAF)<0.005.

**Table 1:** Characteristics of the individuals in the TUK1 and TUK2 samples.

	TUK1	TUK2
N total	203	210
Age, years	60.11 (9.01)	56.50 (10.72)
BMI, kg/m^2^	26.61 (5.07)	25.32 (4.15)
**Sensitive individuals**		
N	101	114
HPT, C^0^	43.09(2.47)	42.44(2.32)
HPST, C^0^	44.27(1.10)	43.95(1.54)
**Insensitive individuals**		
N	102	96
HPT, C^0^	48.17(0.72)	47.43(0.98)
HPST, C^0^	49.78(0.28)	49.37(0.41)

The mean (standard deviation) is shown for the TUK1 and TUK2 samples.

N represents sample size; BMI, body mass index; HPT, heat pain threshold; HPST, heat pain suprathreshold.

**Table 2:** Details of the SNVs identified in TUK1 and TUK2 samples.

Functional Effects	TUK1	TUK2
Mb sequenced (number)	32	44
number of exons (k)	180	300
nonsynonymous coding	60,353	82,293
partial codon	4	3
splice site	8,155	11,060
stop gained	1,100	1,728
stop lost	76	124
synonymous coding	44,878	56,993

The number of SNVs detected is shown according to their functional consequences, for the TUK1 and TUK2 samples.

**Figure 1:**
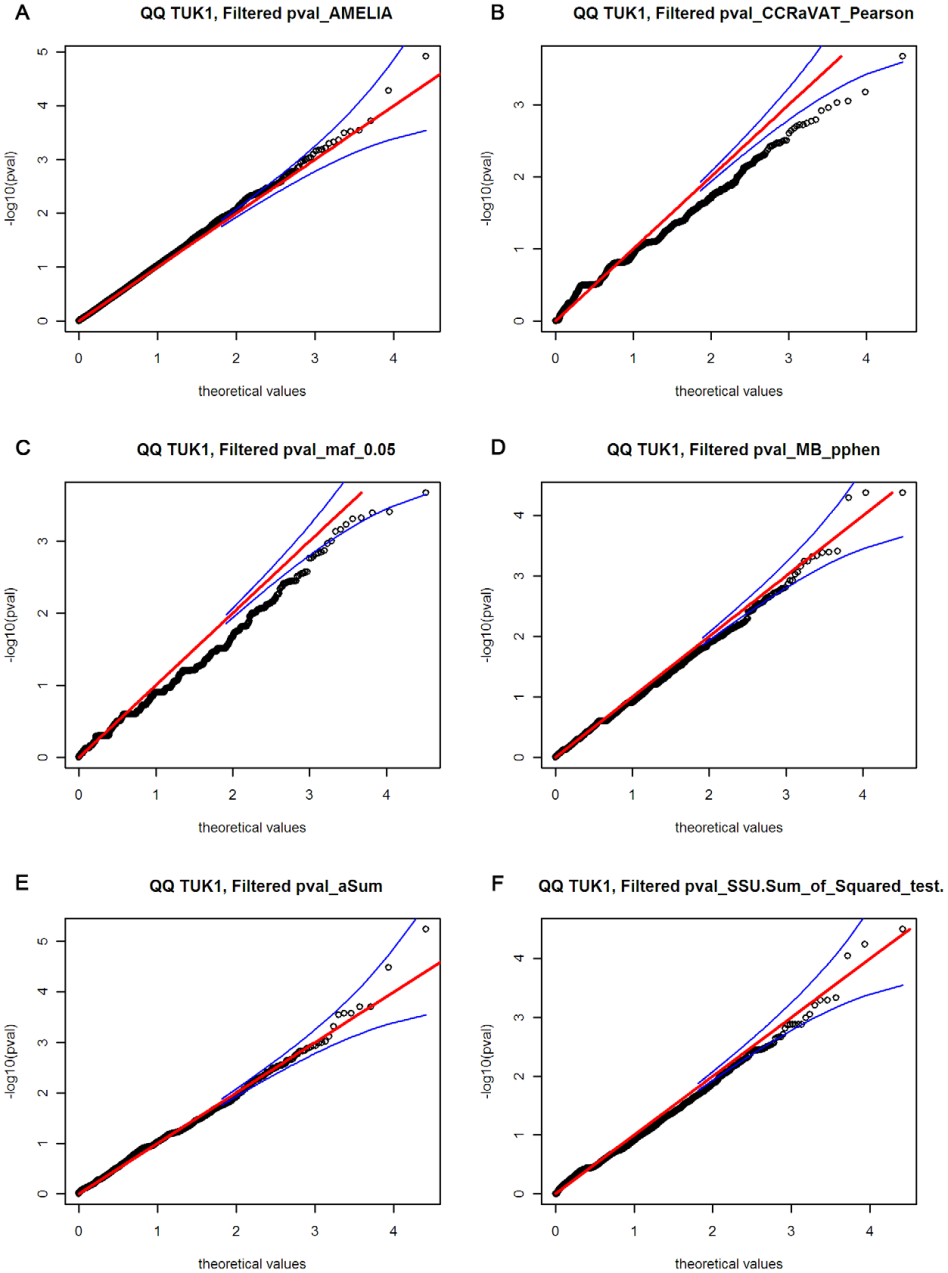
Quantile–quantile plots for the six different variant burden analysis methods. Quantile–quantile plots are shown for: (a) AMELIA, (b) CCRaVAT, (c) fixed filter test, minor allele frequency <0.05, (d) Madsen-Browning with polyphen weights, (e) Han and Pan aSumtest, (f) SSU, sum-of-squares test (Han and Pan).

**Figure 2:**
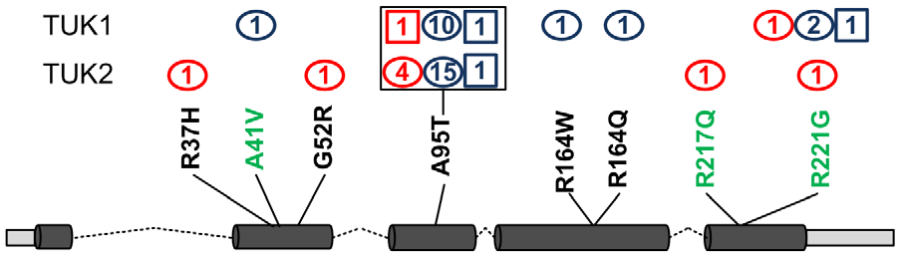
SNVs identified in gene *GZMM*. Schematic showing number of subjects in TUK1 (top row) and TUK2 (bottom row) having nonsynonymous SNVs within the *GZMM* gene, with novel variants in black and those described in dbSNP in green. Subject counts in blue are for pain insensitive subjects and in red, pain sensitive. Squares represent homozygous and ovals heterozygous mutations. Exons are shown as dark cylinders, UTRs pale grey rectangles and introns dotted line.

Results of comparison between the 21 tests for analysing rare variant association were represented using a heat map (correlation matrix, Table S2). As expected, methods having similar underlying assumptions provided highly correlated results and show “hot” on the heat map. A pair of tests was selected from each category/correlation block based on the correlation matrix and their QQ plots (Figure 1) giving 6 gene-centric variant burden tests employed in the final analysis. Using these 6 methods we identified genes containing variants associated with pain sensitivity, shown in Table 3 ranked by strength of evidence. Of the 20,038 exonic gene regions tested, 17,129 (from 14,109 unique genes) gave consistently non-missing p-values across the 6 selected variant burden tests. The p-value considered significant under Bonferroni correction that would apply for a single set of tests based on this number of genes was p<3.0e-06: no variants passed this threshold so none could be considered unequivocally associated with pain sensitivity. The gene *GZMM* was the most highly associated with heat pain sensitivity, p = 6.86e-05 in the combined TUK1 and TUK2 analysis. Variants identified in *GZMM* are shown in Figure 2. For SNV A95T there were 12 alleles (1×2+10) in the heat insensitive vs 1 in heat sensitive (p = 0.005, by Fisher's exact test) in TUK1. While in TUK2 we found 17 alleles in the heat insensitive vs 4 alleles in the heat sensitive (p = 0.0016). Individuals insensitive to heat pain manifested rare variants more frequently than the sensitive, across *GZMM* (Figure 2). Finally, the distribution of variants differed between the pain insensitive and sensitive groups, with the pain insensitive showing a relative enrichment of rare variants (Figure S2).

**Table 3:** Genes associated with heat pain sensitivity using six methods of gene-centric variant burden analysis.

Gene	List Source	Evidence category	Chr	Gene annotation	Primary list 2nd lowest p-value	Merged 2nd lowest p-value
*GZMM*	Primary TUK1&2	Very high	19	granzyme M (lymphocyte met-ase 1)	0.00010	6.86×10^−05^
*CCNJL*	Primary TUK1&2	High	5	cyclin J-like	0.00010	0.00025
*ZNF767* *	Primary TUK1&2	High	7	zinc finger family member 767	0.00036	0.00070
*LAMA4*	Primary TUK1&2	High	6	laminin, alpha 4 [Homo sapiens]	0.00041	0.00117
*OR5F1*	Primary TUK1&2	High	11	olfactory receptor, family 5, subfamily F, member	0.00074	0.00033
*TBK1*	Primary TUK1&2	High	12	TANK-binding kinase 1	0.00083	0.00030
*DDAH1*	Primary TUK1&2	High	1	dimethylarginine dimethylaminohydrolase 1	0.00165	0.00028
*PDHA2*	Merged dataset	Medium	4	pyruvate dehydrogenase (lipoamide) alpha 2	-	0.00060
*FBXW7*	Merged dataset	Medium	4	F-box and WD repeat domain containing 7	-	0.00063
*DLD*	Merged dataset	Medium	7	dihydrolipoamide dehydrogenase	-	0.00078
*RHEB*	Merged dataset	Medium	7	Ras homolog enriched in brain	-	0.00097
*CCDC111*	Primary TUK1&2	Medium	4	coiled-coil domain containing 111	0.00075	0.00056
*TAGAP*	Primary TUK1&2	Medium	6	T-cell activation RhoGTPase activating protein	0.00075	0.00070
*MYPN*	Primary TUK1&2	Medium	10	myopalladin	0.00149	0.00095

Category of significance (“very high” “High”,“Medium”) as defined below:

* represents known pseudogene; Chr chromosome.

“High” p<0.00044 (based on p-value which reaches genome-wide significance if replicated, based on gene counts); “Very High” means “High” plus the merged data is more significant than by combining TUK1 and TUK2 p-values (implying synergy of direction); “Medium” is p<0.001.

### Pathway analysis results

**Figure 3:**
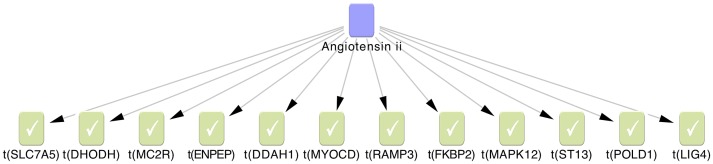
The Angiotensin II regulatory network was identified by causal reasoning from 138 genes associated with pain sensitivity. Causal reasoning uses directed molecular interactions to work upstream from the genes in this study (green) to identify regulators such as angiotensin II (blue) that have a causally correct regulatory role for a significant number of input genes. Correctness is determined by giving each input gene a direction of effect. Here, we presumed a loss of function (e.g. down regulation in activity) to all of our genes. Angiotensin II has direct causal connections to 12 of the genes from our 138, which can be increased to 30 if one intermediary node is allowed in the network (Figure S1). Distribution of novel rare variants identified according to minor allele frequency in a) TUK1 and b) TUK2 datasets.

The 2^nd^ lowest p-value among 6 gene-centric variant burden tests was used as a cut-off to prioritise genes for pathway analysis (see Methods, statistical analysis). After merging TUK1 and TUK2 datasets, we identified 138 unique genes harbouring a rare variant with a 2^nd^ lowest p-value<0.01. First we examined the functional annotations of these 138 genes using the online functional annotation tool DAVID (http://david.abcc.ncifcrf.gov/) [10]. Nine high level GO terms were nominally significantly enriched in the gene list eg. “plasma membrane” and “intracellular signalling cascade”. None reached significance after multiple testing correction or offered obvious insights into mechanisms of altered pain sensitivity (results not shown). We applied causal reasoning to our data [11], which uses a large curated database of directed regulatory molecular interactions to identify the most plausible upstream regulators of a gene set. Of the 138 genes 86 were present in our database of causal interactions, from which we identified 4 nominally significant regulatory networks (Table 4). One of the regulatory networks, angiotensin II (Figure 3), was highly enriched for a pain signal with 12 out of 204 genes in the network also in the set of 86 genes with a nominal genetic burden. This yields an odds ratio of 7.6, an enrichment p = 3.4×10^−7^ and a correctness p = 1.2×10^−8^. Since 1108 pathways were tested, this adjusts to enrichment p = 3.8×10^−4^ and correctness p = 1.4×10^−5^ under multiple test correction.

**Table 4:** Pathways identified by causal reasoning.

Pathway	Correctness p (Bonferroni corrected p)	Enrichment p (Bonferroni corrected p)	No. connections (no. possible connections)	Type of pathway
Angiotensin II −	1.2×10−8 (1.4×10^−5^)	3.4×10−7(3.8×10^−4^)	12 (204)	Peptide
Estrogen −	0.001 (>1)	0.002 (>1)	3 (26)	Biological process
Adipocyte differentiation −	0.004 (>1)	0.005 (>1)	4 (76)	Biological process
Triamcinolone acetonide +	0.04 (>1)	0.01 (>1)	3 (94)	Chemical

Causal reasoning [11] uses a large curated database of directed regulatory molecular interactions to identify the most plausible upstream regulators of a gene set with a proposed directionality (eg. down-regulated). We considered the 138 genes identified to contain loss of function mutations. One regulatory pathway (angiotensin II) is significant after correction for multiple testing when considering directionality (Correctness p) as well as when ignoring directionality of regulation (Enrichment p).

The sign (−/+) after the regulator's name indicates the loss (−) or gain (+) of activity required to explain the loss of function mutations.

Enrichment p-value indicates the significance of the number of connections apparent in our data compared to the total number of connections.

Correctness p-value also accounts for the regulatory direction (+/−) and indicates the significance of the hypothesis as a regulator.

We also investigated whether the genes identified were known to interact physically with proteins playing a role in pain. For this we used the BioGrid database of protein-protein interactions. Notable connections included the binding of synaptotagmin-9 (SYT9), a membrane trafficking protein activated by calcium, to TRPV1, the capsaicin receptor, which plays a key role in thermal nociception [12]. The extracellular matrix glycoprotein laminin B1 chain (LAMB1) interacts with the voltage dependent calcium channel Cav2.1 (CACNA1A) [13]. The receptor activity modifying protein 3 (RAMP3) binds to the calcitonin receptor (CALCRL), for transport to the membrane. Here the calcitonin receptor recognises the calcitonin gene related peptide (CGRP), a hormone proposed to contribute to pain transmission and inflammation [14]. Finally, the sodium-hydrogen exchanger regulatory factor 1 (SLC9A3R1), binds the beta-2-adrenergic receptor (ADRB2) [15], nitric oxide synthase 2 (NOS2) [16], membrane metallo-endopeptidase (MME) [17] and the opioid receptor kappa 1 (OPRK1) [18].

## Discussion

Patients with chronic pain have increased sensitivity to noxious stimuli such as heat and pressure compared to controls [19] as well as to non-noxious stimuli such as sound [20]. These observations support the notion that the processing of external stimuli is heightened or exaggerated in chronic pain states. Thus, people harbouring gene variants associated with greater sensitivity to heat pain stimulus are thought to be at increased risk of developing chronic widespread pain. The premise of this work was that understanding better the genetic influence on normal pain processing would shed light on the biological pathways underlying the pathology of chronic pain. In this project we adopted novel methods - biotechnological and statistical - to identify rare sequence variation contributing to pain sensitivity in normal individuals. The advent of high throughput genotyping technologies has helped to unravel the aetiology of many complex diseases and quantitative traits. In particular, genome-wide association (GWA) studies have uncovered many common variants associated with quantitative phenotypes. However, GWA is underpowered to detect association of rare variants, and the common variants identified so far explain only a fraction of the trait heritability. As whole-genome sequencing has become more cost-efficient it is now feasible to examine the effect of rare variants. The hypothesis that multiple rare variants explain a proportion of the missing heritability is gaining more attention [21].

Rare variants with moderate to high penetrance have been associated with a number of extreme phenotypes (summarised in [22]). For quantitative phenotypes, sampling and comparing the extremes of traits has become an accepted strategy for identifying disease-causing rare variants in exome sequencing [23]. In this novel exome project of pain perception in normal individuals, no genetic variants of large effect were identified. Considering that the statistical power after applying stringent multiple test correction was limited, we can't exclude moderate or small contributions by individual SNVs to the experimental pain phenotype. Indeed, we have noted a differential distribution of rare variants between the pain sensitive and insensitive subjects (Figure S2), which suggests enrichment of multiple SNVs of small effect at the extremes of the normal distribution. This study also provides proof of principle of the utility of the exome sequencing method.

Such an approach has been used successfully in the, albeit more limited, setting of sequencing ion channel genes in epilepsy [24]. The authors highlighted the need for cell and network analysis to optimise information obtained from such a study. A variety of statistical methods have been developed for analysis of association of rare variants with complex traits, but there remains a paucity of data regarding the genetic architecture underlying complex traits such as pain perception. For this reason we elected to use a variety of tests based on different underlying assumptions so that no rare variant associated with pain perception would be missed.

*GZMM* was the only gene classified as having “very high” evidence of association to thermal nociception (Table 3 and Figure 2: see Methods: statistical analysis for classification definitions). It encodes granzyme M, one of the serine proteases produced and stored in the granules of immune cells such as lymphocytes and natural killer cells [25]. While we could not find reports of association with pain in the literature, granzymes are known to play an important role in apoptosis [26] and in the initiation of inflammation: elevated levels have been detected in rheumatoid synovial fluid [27] and granzyme B expression increased in lesional atopic dermatitis skin [28]. In the “high” evidence category, the enzyme encoded by the seventh gene, *DDAH1*, plays a role in nitric oxide generation by regulating cellular methylarginine concentrations, which in turn inhibit nitric oxide synthase. Although both anti-nociceptive and pro-nociceptive roles of NO have been reported, overproduction of NO - together with free radicals - contribute to central sensitisation and the pathogenesis of abnormal pain states via association with NMDA receptor mediated signalling events. In support of this, circulating NO has been shown to be elevated in chronic widespread pain patients [29]. The links between pain and other genes listed in Table 3 (such as *CCNJL* and *TBK1*) are tenuous at present.

To explore further the interplay between the SNV-containing genes identified we applied causal reasoning, an algorithm using directed molecular relationships between biological entities to identify up-stream regulators of a set of input genes [30]. We identified 4 regulatory networks that were nominally significant, one of which (angiotensin II) remained significant after correction for multiple testing (correctness p = 1.4×10^−5^, enrichment p = 3.8×10^−4^). Angiotensin II is a peptide hormone involved in the control of blood pressure. This network connected 12 of our identified genes into a causal network (Figure 3). Angiotensin II has been already been implicated in central pain: it has been shown to facilitate pain-related behaviours in experimental animals [31] including responses to thermal stimuli similar to those employed in the current studies. The mechanism appears to be via the modulation of descending brainstem pathways. Blocking the receptors for angiotensin II (so called AT1 receptors) reverses some pain-related behaviours in models of chronic pain, suggesting a role for endogenous angiotensin II. For example, AT-1 receptor antagonist telmisartan has been shown to abrogate pain in the sciatic nerve constriction model in rats [32]. The data from several small clinical studies in humans have been conflicting [33], [34] but a recent phase II clinical trial of a AT2 receptor antagonist (AT2 receptors are expressed by primary afferent nociceptors) found a significant improvement in the pain of a group of patients with post-herpetic neuralgia (http://www.spinifexpharma.com.au/DRUG-DISCOVERY.html).

Our causal reasoning analysis allowed for only one interaction upstream of the genes in our dataset to be included. However, allowing two interactions increased the number of genes from this study that may be causally linked to angiotensin II to 30 genes. Angiotensin II can also be causally linked to known pain relevant processes. For example, *PTGS2*, the gene encoding cyclooxygenase 2 (COX-2, the target of the non-steroidal anti-inflammatory drugs) is regulated by angiotensin II [35]. COX-2 produces prostaglandin E2 (PGE2), which is released in damaged or inflamed tissues and binds to nociceptive nerve terminals via PGE2 receptors (so called EP receptors), leading to cAMP production. This leads to post-translational modification of several target proteins within nerve terminals that regulate nociceptor excitability, including voltage-gated sodium channels [36]. The current study using novel exome sequencing methods supports the notion that the angiotensin II pathway is important in pain regulation in man and suggests that genetic variation in the pathway may influence sensitivity to heat pain, at least in the Northern European population.

A third form of analysis examined the target genes in a network of all human protein-protein interactions from the BioGRID database. We asked if any of the proteins encoded by the genes identified in this study were known to interact directly with proteins having a role in pain. We found known physical interactions with several pain-relevant proteins including ion channels (TRPV1 and Cav2.1), the CGRP receptor and the kappa opioid receptor. It is clear therefore that although we did not identify any genes immediately associated with nociception, several play key roles in processes linked to the reception and transduction of pain signals by their physical and biochemical interactions with important pain mediating complexes.

This study highlights the potential of using a combination of sophisticated analytical methods to identify associations underlying rare variants in quantitative traits. While the predicted effect sizes are relatively small and require large samples, we have made progress in understanding the genetic architecture underlying heat pain sensitivity. Despite recent advances in both DNA sequencing technology and the statistical methods to analyse such complex datasets, the identification and follow-up of associations of individual gene variants remains a challenge. Our results lend weight to the notion that angiotensin II plays in important role in signal transduction in pain and this pathway merits further biological investigation.

## Materials and Methods

Ethics committee approval was obtained from Guy's and St Thomas' Hospital research ethics committee. All subjects were volunteer singleton members of female monozygotic (MZ) and dizygotic (DZ) twins from the TwinsUK register of King's College London [37]. Thus we did not perform a classical twin study and did not need to adjust for relatedness. QST was performed according to standard methods (see Supporting information) in which measures of heat pain threshold (HPT) and heat pain suprathreshold (HPST) were made.

### Selection for exome sequencing

HPST score was selected as the primary metric because reproducibility was greater (intra-class correlation coefficients, HPST = 0.59 (0.51, 0.68); HPT = 0.34 (0.23, 0.46)). HPST was also found to have greater heritability (HPST h^2^ = 0.44; HPT h^2^ = 0.29). The two phenotypes were correlated (r = 0.64). To select subjects who were relatively pain sensitive/insensitive for exome sequencing, the following protocol was adopted: a subject was included only if their HPT score was in the same half of the distribution as the HPST and, in the case of MZ twin pairs, the co-twin also resided in the same HPST tail. For DZ twins, the entire pair was excluded if they fell into opposite tails; if both were in the same tail, the more extreme twin was selected. In no case were two members from a twin pair selected. In addition, three samples provided by HapMap were analysed twice – in TUK1 and TUK2 – to enable comparison of the methods. Additional detail is provided in Text S1.

### Exome sequencing

DNA extracted from whole blood was sent to BGI for exome sequencing [38]. The qualified genomic DNA sample was randomly fragmented by Covaris technology with resultant library fragments 250–300 bp. Adapters were ligated to both ends of the fragments. Extracted DNA was amplified by ligation-mediated PCR (LM-PCR), purified and hybridized to the NimbleGen human exome arrays for enrichment; non-hybridized fragments were then washed out. The target enrichment of the TUK1 samples were performed using hybridization to the NimbleGen 2.1 M array, while the shotgun libraries of the TUK2 samples were enriched using NimbleGen EZ v2 library. The captured LM-PCR products were subjected to quantitative PCR to estimate the magnitude of enrichment. Each captured library was then loaded on Illumina platforms and high-throughput sequencing was performed on each library. The BGI used Illumina GAIIx for sequencing of the TUK1 samples and a Hiseq2000 platform for TUK2 samples. Raw image files were processed by Illumina base-calling software v1.6 (and v1.7), and the sequences of each individual were generated as 75 bp (and 90 bp) paired-end reads for TUK1 (and TUK2) sets respectively. The fastq files were generated from the raw data after removing the adapters and low quality reads.

### Exome mapping

Both datasets were mapped to the NCBI Human Reference (GRCh37; hg19) using BWA v0.5.5 (v0.5.9). We considered the default parameter –q 15 for read clipping, and a maximum insert size of 600 bp for proper pairing of the short reads. The alignment files for each lane were sorted and indexed by SAMtools [39] before constructing the library-level bam files. We also tried to improve the accuracy of the base quality scores by running a recalibration stage using Genome Analysis Toolkit (GATK) v1.0.5777 [40]. On average 5% of each library was contaminated with duplicate fragments, which were removed before variant calling. An extra step of local re-alignment was applied only to the TUK2 data to improve the sensitivity and specificity of mismatches near indel sites.

For quality control (QC) of the TUK1 data, we studied the histogram of depth distribution, the distribution of inferred insert sizes in the bam files, the GC content distribution for reads mapped to forward and reverse strands, the depth of coverage as a function of percentile of unique sequences ordered by GC content, and the fraction of each chromosome covered by the exomes. The distribution of per-base sequencing depth for each sample was evaluated as was the cumulative depth distributions in target regions, and sequencing depth and coverage of the target region per chromosome. The TUK2 dataset had a slightly higher depth of coverage over the capture target region (CTR), with average 71× depth (compared to 69× for TUK1), whereas average coverage of the CTR was 97.5% for TUK2 (compared to 96.5% in TUK1). In the TUK1 panel we discarded and re-sequenced a few lanes, which showed very low target coverage; hence requiring all the exomes to cover more than 70% of the CTR by at least 20× in both datasets. We observed that although the mean depth was comparable, the fraction of CTR covered at a given depth was generally lower in TUK2 set, e.g. CTR coverage at ≥20× was 80.3% for TUK2 compared to 89.1% for TUK1. This alludes to the greater coverage uniformity of the 2.1 M array compared with that of the solution-based EZ sequence capture.

### Variant calling

For TUK1, we ran SAMtools v0.1.8 ‘pileup’ while limiting maximum depth for indels to 500. Then we filtered the SNVs (with ‘varFilter’) with SNV and indel Phred-scale quality scores less than 20, and minimum and maximum depth at 8 and 300 respectively. The GATK v1.0.5777 was run for TUK1 using default values and a minimum confidence threshold 30 and minimum read mapping quality at 10. We subsequently filtered the GATK SNVs by keeping only those with alternative allele quality score ≥ 20 and depth within [8,300] interval. For TUK2, we ran SAMtools v0.1.16 ‘mpileup’ together with ‘bcftools’ using default parameters, but requiring the SNV quality score and depth interval to satisfy the same criteria of the TUK1 set (i.e. QUAL≥20 and 300≥DP≥8). The GATK calling for TUK2 data followed the same procedure as for TUK1. We further filtered all the variants outside the capture target region. Overlapping results SAMtools and GATK were extracted. The discordance (about 5%) was largely attributed to unique calls, however we observed a small fraction (less than 1%) of SNVs called by both algorithms were assigned mismatching genotypes (homozygous non-reference vs heterozygous). We ran the GATK on the coordinates of the overlap to determine the non-variant genotypes hence adjusting the missing rates. The single-sample variant files were then merged (using ‘merge-vcf’) into two large variant call files (VCF) each containing the entire sample variants. Table S1 compares the SNV statistics for TUK1 and TUK2 samples.

### Genotype QC

We evaluated the genotype concordance between the exome and pre-existing GWAS datasets. We observed greater agreement between GWAS and TUK2 (average 99.8%) than GWAS and TUK1 (99.3% concordance) (Table S1). Three samples were identified as highly discordant with GWAS (52%, 54% and 51% rates). A multi-dimensional clustering analysis of these three exomes together with the entire GWAS dataset for 5,654 twins, confirmed that they were true outliers so were excluded from statistical analysis. Duplicate samples in TUK1 allowed estimation of genotype error rate. Out of ∼35 M bases on the 2.1 M array which had been genotyped, 295 and 374 sites were discordant between duplicates. This sets a type 1 error rate for genotyping of approximately 1.0e-05, or 0.001%.

### Statistical analysis

The wide variety of methods to analyse rare variants generally fall into three broad categories: “collapsing” methods, which test for differences in rare variant accumulation; “carrier-based” tests, which test for differences in the number of subjects carrying a certain class of variant (usually at least partially based on frequency thresholds); and “multivariate” tests, which test for differences in variant patterns, and is further subdivided into kernel-based and regression-based methods. Using several tests from each category we ran 21 different gene-centric variant burden tests on the TUK1 set and the results correlated (and displayed as a “heat” map, Table S2). A pair of tests was selected from each category/correlation block based on the correlation matrix and the QQ plots (Figure 1). The six statistical methods selected for this project were:-

- AMELIA: Allele Matching Empirical Locus-specific Integrated Association test. Multivariate test considering both common and rare variants, and is based on genotypic similarity rather than rare allele accumulation [41]
- aSum: Data adaptive sum test. A regression based collapsing approach, which takes account of the direction of effect of the alleles. This type of method is expected to tolerate misclassification eg. if alleles with different functions are collapsed together [42]
- SSU (Sum of Squares Test): a test analogous to traditional multivariate analysis on a binary trait [42]
- simple threshold test: a case/control by subject on carriers with one or more variants having MAF<0.05. It is similar to the CAST method [43]
- CCRaVAT (using Pearson test): collapsing method examining the accumulation of rare alleles using analysis of contingency tables. Like ARIEL, it is sensitive to linkage disequilibrium, however it evaluates the presence or absence of individual rare alleles in cases or controls (rather than the proportion rare variants) [44]
- Madsen and Browning using polyphen weights (MB pphen): method combines variants by weighting based on allele frequency and, optionally, polyphen predictions (selected here) [45]

A primary list of genes harbouring rare variants was drawn up based on combining the p-values from TUK1 and TUK2 sets using Fisher's method. To identify signals from genes with concordant variant patterns across TUK1 and TUK2 datasets, the top genes from the merged raw TUK1 and TUK2 datasets were also considered as relevant signals. This combination did not comprise the primary list because the TUK1 and TUK2 sequencing were performed on different capture platforms: some regions did not overlap between the two. Further details are provided in Supporting information.

In addition to the issue of combining TUK1 and TUK2 was the challenge of combining and sorting the results of the 6 gene-centric variant burden tests which were relatively new and not well understood. Because the 6 tests comprised 3 pairs of similar test methods (Table S2) we considered that a result was not robust if it was significant for only 1 test category. Significance in more than 1 category added confidence that a result was less likely to be a false signal. To prioritize genes that were either significant in more than one category or consistently significant for both tests within a pair, we prioritized genes based on the 2^nd^ lowest p-value from the 6 selected tests. This approach also ensured that the top gene list could not be dominated by anomalies from a single test. Significant results using the 2^nd^ lowest p-value were obtained in two ways: from combining the TUK1 and TUK2 p-values via Fisher's formula, and by merging the datasets (Table 3). A gene was classified as “High” evidence if its 2^nd^ lowest p-value achieved p<0.00044 (the p-value such that replication would achieve a genome-wide significant meta-analytic p-value), and “Very High” if this occurred with the combined dataset being more significant than the combination of the p-values across the two halves. “Medium” priority was given any gene which achieved p<0.001 for its 2^nd^ lowest p-value in either the merged dataset or the combination of the p-values across the two halves.

### Pathway analysis

After removing genes showing an opposite direction of effect and after merging the datasets, we identified 138 unique genes having a 2^nd^ lowest p-value<0.01. These were considered for more detailed analysis. We looked first for enriched Gene Ontology categories within these genes using DAVID [10] with an EASE p-value<0.05. Then we undertook causal reasoning [11] which uses a large curated database of directed regulatory molecular interactions to identify the most plausible upstream regulators of a gene set. Consequently it allows the recapitulation of regulatory networks/pathways associated with genes of interest. The method offers two measures of statistical significance. The enrichment p-value corresponds to a standard gene set enrichment test on the set of downstream genes, whereas the correctness p-value takes the direction of regulation into account. For the latter, each associated gene was considered as a down-regulated transcript in the causal reasoning network ie. assumed loss-of-function mutations. As a background set for the significance calculations we considered the intersection of the set of all genes covered in either the TUK1 or TUK2 study and all transcripts in our causal reasoning database. This set consists of 9275 genes. A regulatory hypothesis was considered nominally significant with a p-value<0.05 and significant at a 0.05 level after application of the Bonferroni correction for multiple testing. As we are considering 1108 potential upstream regulators in the underlying database, a Bonferroni corrected p of 0.05 corresponds to a nominal p-value of 4.5×10^−5^. Finally, we searched for direct physical interactions between proteins identified in this study and proteins known to have a role in pain using protein interaction data from the BioGrid database [46].

## Supporting Information

Figure S1 Relative proportions of novel and recognised variants in the two samples. Recognised variants were defined by their presence in dbSNP. Data are shown by allele frequency (y axis) for (a) TUK1 dataset and (b) TUK2 dataset.
(TIF)

Figure S2 Relative frequencies of novel rare variants detected, by pain sensitivity. The frequency distribution of nonsynonymous rare variants (MAF- minor allele frequency <5%) for the most significantly associated 32 genes identified, by pain sensitivity: pain insensitive (red bars) and pain sensitive (green bars). Pain insensitive individuals harboured more rare variants than the pain sensitive: pain sensitive variant counts were 0.51 (95% CI: 0.272–0.962) that of the insensitive. As such a finding could result from a few one-sided genes we also adjusted for variant excess differing by gene. There remained a small excess of variants in pain insensitive individuals across all genes, p = 0.033. Seventeen genes had at least 10% difference in rare variant counts (MAF<0.05) between the sensitive and insensitive subjects. Of these 17 genes, 14 had excess insensitive subjects, while only 3 had the excess sensitive subjects (p = 0.0127 for 2-sided t-test).
(TIF)

Table S1 Details of exome sequencing. Descriptive statistics for variants identified in exome sequencing of TUK1 and TUK2 sample sets. The values were generated from filtered variants for all samples which passed QC.
(DOCX)

Table S2 Relationship between the 21 rare variants analysis methods used in the TUK1. Heat Map or correlation matrix of the −log10 p values (pval) for each pair of 21 methods run in TUK1. Higher correlations are colored red, lower correlations are colored blue. The 6 tests selected for use in the study are marked by italics and underlined. The full set of 21 analytical methods are as follows: KBAT represents kernel based association test; AMELIA, Allele Matching Empirical Locus-specific Integrated Association; ARIEL, Accumulation of Rare variants Integrated and Extended Locus-specific test; CCRaVAT, Case-Control Rare Variant Analysis Tool; FishExc, Fisher's exact test; pval maf 0.01, p value of fixed threshold test with minor allele frequency <0.01; MB, Madsen and Browning weighted approach; VT, variable threshold; pphen, Polyphen; SSU, sum of squared test; aSum permuted, Han and Pan's aSum test permuted; aSum, Han and Pan's aSum test.
(DOCX)

Text S1 Further details are provided regarding the subjects and their selection, the quantitative sensory testing, and rare variant analysis.
(DOCX)
