# Supplementary figures and images for: Genes Contributing to Pain Sensitivity in the Normal Population: An Exome Sequencing Study
Source: PLoS Genet. 2012 Dec 20;8(12):e1003095. doi: 10.1371/journal.pgen.1003095 (PMC3527205; doi:10.1371/journal.pgen.1003095)

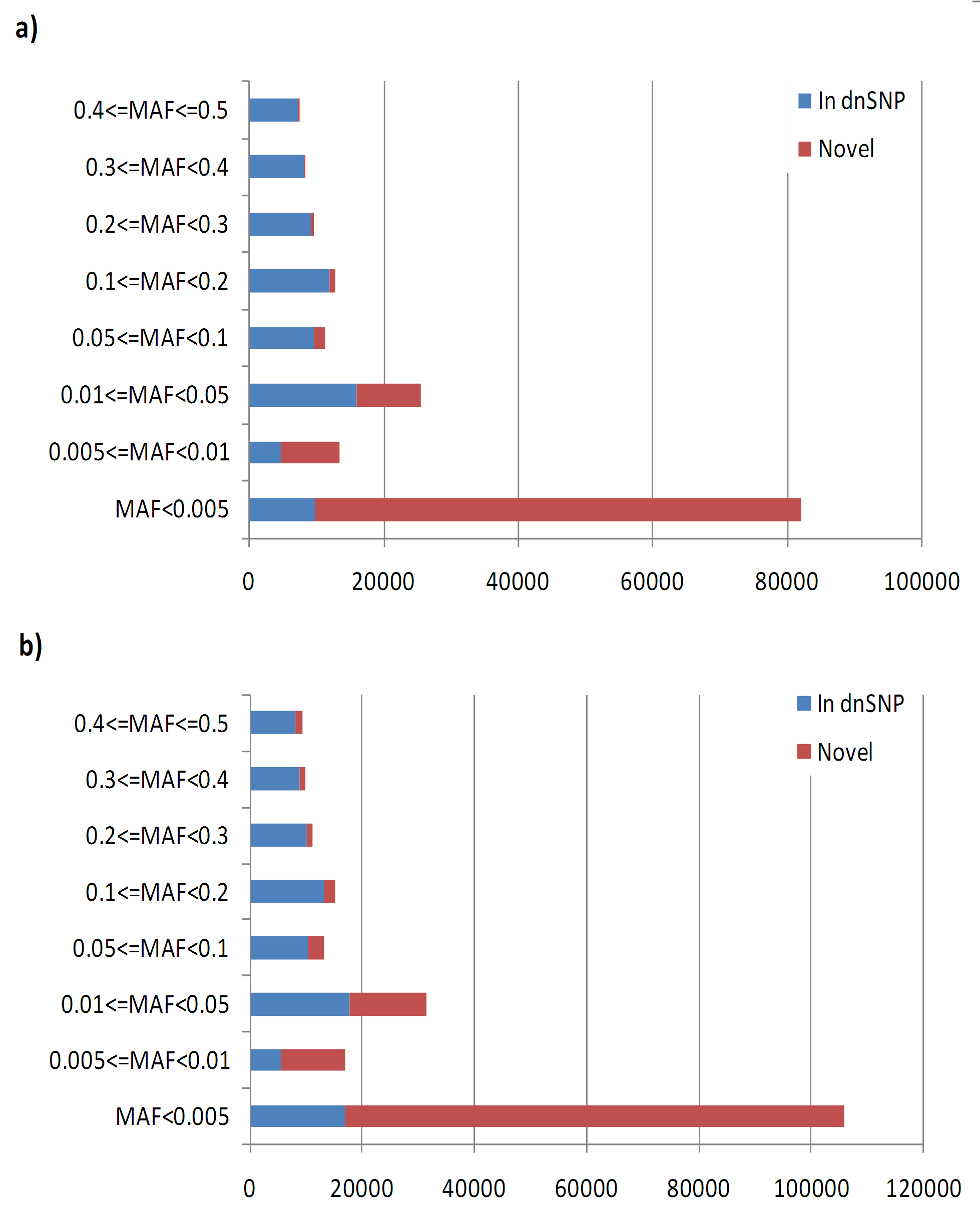

Supplement: Figure S1 — Relative proportions of novel and recognised variants in the two samples. Recognised variants were defined by their presence in dbSNP. Data are shown by allele frequency (y axis) for (a) TUK1 dataset and (b) TUK2 dataset. (TIF) [file pgen.1003095.s001.tif]

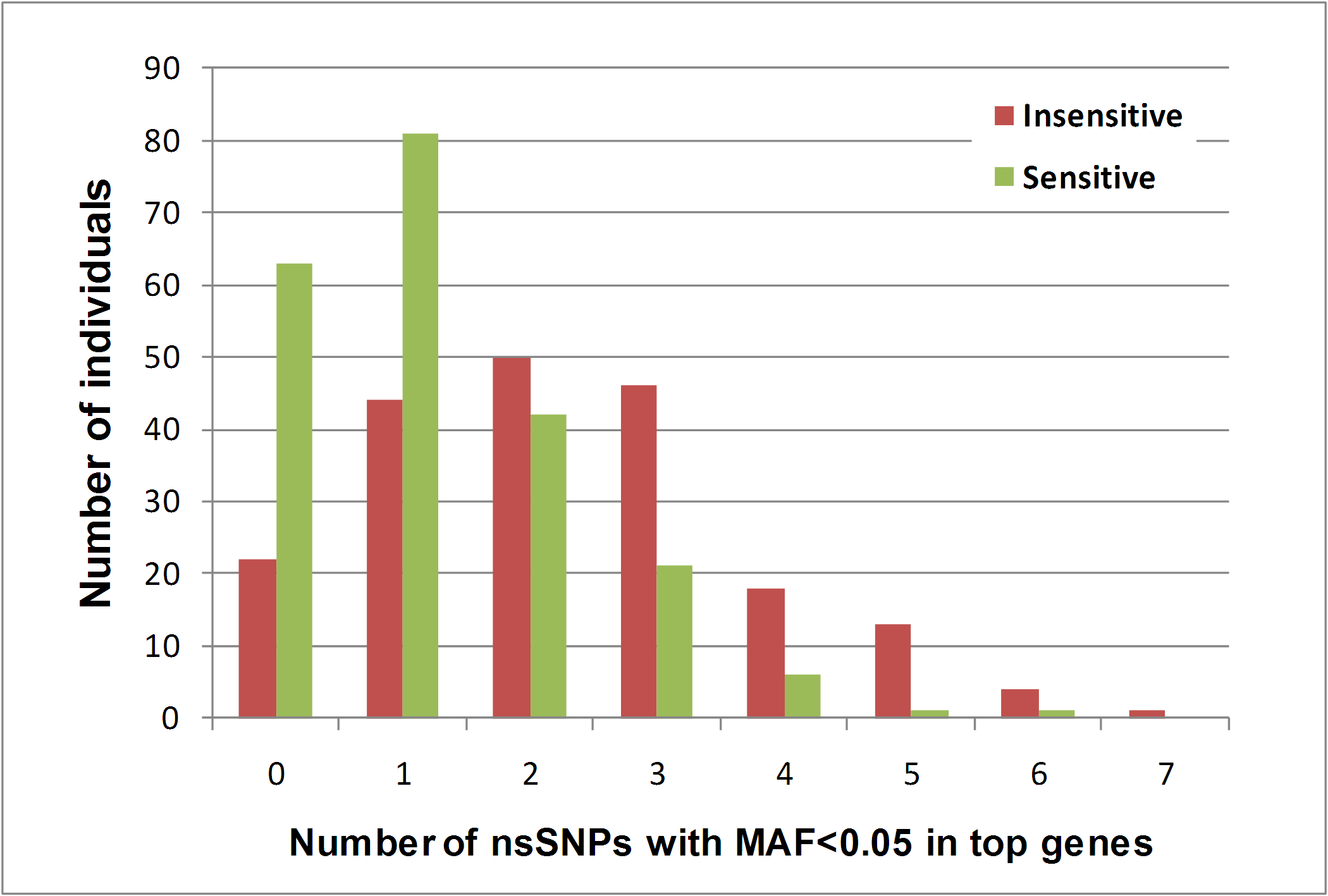

Supplement: Figure S2 — Relative frequencies of novel rare variants detected, by pain sensitivity. The frequency distribution of nonsynonymous rare variants (MAF- minor allele frequency <5%) for the most significantly associated 32 genes identified, by pain sensitivity: pain insensitive (red bars) and pain sensitive (green bars). Pain insensitive individuals harboured more rare variants than the pain sensitive: pain sensitive variant counts were 0.51 (95% CI: 0.272–0.962) that of the insensitive. As such a finding could result from a few one-sided genes we also adjusted for variant excess differing by gene. There remained a small excess of variants in pain insensitive individuals across all genes, p = 0.033. Seventeen genes had at least 10% difference in rare variant counts (MAF<0.05) between the sensitive and insensitive subjects. Of these 17 genes, 14 had excess insensitive subjects, while only 3 had the excess sensitive subjects (p = 0.0127 for 2-sided t-test). (TIF) [file pgen.1003095.s002.tif]

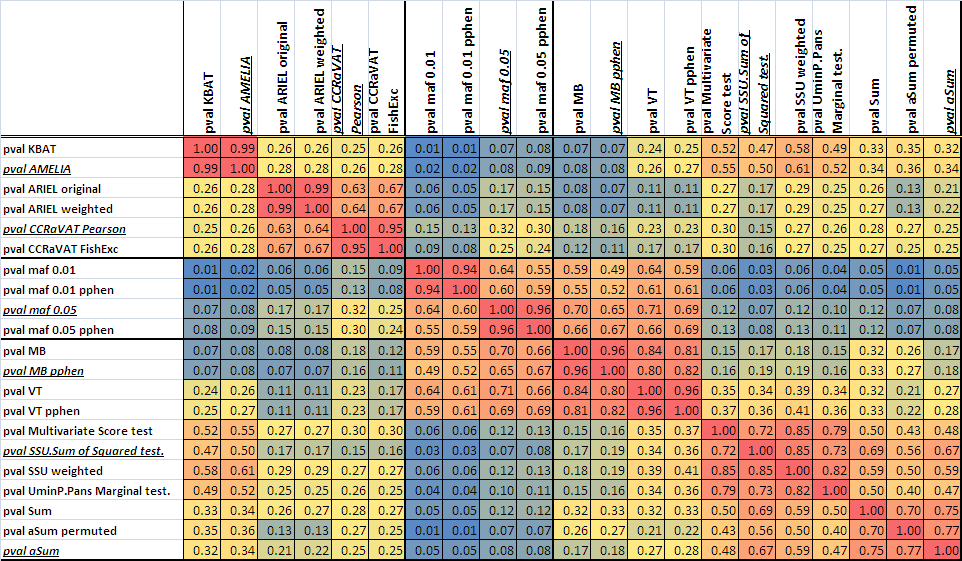

Supplement: Table S2 — Relationship between the 21 rare variants analysis methods used in the TUK1. Heat Map or correlation matrix of the −log10 p values (pval) for each pair of 21 methods run in TUK1. Higher correlations are colored red, lower correlations are colored blue. The 6 tests selected for use in the study are marked by italics and underlined. The full set of 21 analytical methods are as follows: KBAT represents kernel based association test; AMELIA, Allele Matching Empirical Locus-specific Integrated Association; ARIEL, Accumulation of Rare variants Integrated and Extended Locus-specific test; CCRaVAT, Case-Control Rare Variant Analysis Tool; FishExc, Fisher's exact test; pval maf 0.01, p value of fixed threshold test with minor allele frequency <0.01; MB, Madsen and Browning weighted approach; VT, variable threshold; pphen, Polyphen; SSU, sum of squared test; aSum permuted, Han and Pan's aSum test permuted; aSum, Han and Pan's aSum test. (DOCX) [file pgen.1003095.s004.docx]
