# Supplementary material for: Genes Contributing to Pain Sensitivity in the Normal Population: An Exome Sequencing Study
Source: PLoS Genet. 2012 Dec 20;8(12):e1003095. doi: 10.1371/journal.pgen.1003095 (PMC3527205; doi:10.1371/journal.pgen.1003095)
Supplement: Table S1 — Details of exome sequencing. Descriptive statistics for variants identified in exome sequencing of TUK1 and TUK2 sample sets. The values were generated from filtered variants for all samples which passed QC. (DOCX) [file pgen.1003095.s003.docx]

|  | **TUK1** | **TUK2** |
| --- | --- | --- |
| SeqCap Exome array | 2.1M (35Mb, ~20K genes) | EZ v2 (44Mb, ~30K genes) |
| Mean depth on CTR | 69X | 71X |
| Average coverage of CTR | 96.50% | 97.50% |
| Transitions/Transversions | 2.78 | 2.42 |
| Average GWAS Discordance | 58/8,711=0.007 | 22/10,085=0.002 |
| Average SNVs per sample | 23,592 | 31,501 |
| Total SNV on CTR | 160,124 | 226,094 |
| Novel SNV | 89,397 | 132,875 |
| Mean missing rate | 0.029 | 0.078 |
| Average singletons | 277 | 396 |
| Unrelated QC’ed cases/controls | 102 insensitive, 101 sensitive | 96 insensitive, 114 sensitive |
