# Supplementary material for: Genes Contributing to Pain Sensitivity in the Normal Population: An Exome Sequencing Study
Source: PLoS Genet. 2012 Dec 20;8(12):e1003095. doi: 10.1371/journal.pgen.1003095 (PMC3527205; doi:10.1371/journal.pgen.1003095)
Supplement: Text S1 — Further details are provided regarding the subjects and their selection, the quantitative sensory testing, and rare variant analysis. (DOCX) [file pgen.1003095.s005.docx]

**Text S1**

The TwinsUK registry contains twin volunteers recruited through national media campaigns and from other twin registers. They are not selected for any particular trait. Subjects on the register take part in studies that cover a wide range of traits and common medical conditions and in general are not aware of the precise hypothesis being tested. The study was approved by the St Thomas’ Hospital research ethics committee, and all participants provided written informed consent. Twins from this registry have been shown to be comparable to the age-matched general population singletons for a broad variety of medical and behavioural traits [[1](#_ENREF_1)]. Subjects selected for this study had previously been genotyped using Illumina platforms (317k and 610k) and the twins broad contribution to collaborative GWA studies attests to their representativeness [[2](#_ENREF_2),[3](#_ENREF_3),[4](#_ENREF_4)]. Unselected twins were invited to attend St Thomas’ Hospital where they completed questionnaires gathering demographic information, clinical history and current medications. Exclusions included volunteers who had consumed analgesic medication within 12 hours of the study visit, and those with likely impaired upper limb neurology, eg known neuropathy, previous stroke or chemotherapy. Subjects having common painful conditions such as osteoarthritis were not excluded. Venous blood was taken for DNA extraction after the QST had been performed. Results of the exome sequence were compared with pre-existing GWAS information.

**Quantitative Sensory testing (QST)**

Twins underwent sensory testing individually, with the co-twin excluded from the room while the test was performed. The subject was seated at a table onto which the arm was placed. A 25mm^2^ x 50mm^2^ probe connected to a Modular Sensory Analyzer Thermal Stimulator (Somedic, Sweden) was secured with a fabric-covered band on the volar surface of the forearm. Subjects received standardized instructions before both assessments. The HPT represents the temperature at which the sensation evoked by a thermal stimulus changes from feeling ‘hot’ to feeling ‘painful’ while the HPST records the temperature at which the sensation changes from “painful to unbearable”. HPT was measured by heating the probe (rate of 0.5^0^C/s) from an adaptation temperature of 32^0^C until the subject perceived the stimulus as changing from hot to painful and stopped the experiment by pressing a button, at which point the temperature (equivalent to HPT) was automatically logged and the probe temperature returned to 32^0^C. If the subject reached 50^0^C the machine automatically returned to adaptation temperature to prevent thermal burn. A ‘practice run’ at measuring HPT was followed (10 seconds later), by the true HPT measurement. HPST was determined after the probe had been removed to the opposite arm. The probe was heated from 32^0^C (1^0^C/s) until the subject perceived the stimulus as changing from “painful to unbearable” and stopped the experiment, logging the temperature (equivalent to HPST). If the probe reached 50^0^C the machine automatically returned to adaptation temperature to prevent thermal burn. HPST was assessed without a trial run.

**Rare variant analysis**

We implemented the 21 tests on over 20,038 CCDS genes for TUK1 and TUK2 data separately. These regions correspond to the overlap between the two capture targets where we look for any evidence of replication. For ARIEL we set a minor allele frequency (MAF) cut-off of 5% for collapsing the set of rare variants. For AMELIA we considered an incremental permutation approach, where we permuted the SNPs in each region 1,000 times, after which if p-value remained below 0.01 we increased the iterations to 10,000, and subsequently to 100,000 and 500,000, checking at each stage whether p-value remains below the same threshold. We ran the aSum test using a permutation value of 10,000 and the cut-off parameter, alpha 0=0.1, for code flipping based on the marginal p-value of the SNP. The VT test was run with 1,000,000 permutations, and using PolyPhen probability scores. The PolyPhen predictions for non-synonymous SNPs were generated using PPH software v2. For input probabilities to VT we assumed prob=0.5 for common variants (MAF >=0.01) unless they were stop-gain or splice site variants (prob=1). For rare variants we used the polyphen scores if they exist (non-synonymous), otherwise we assumed prob=0.5 for the rest of rare variants. These scores were further converted to VT weighting before analysis.

Accounting for both the correlation structure and the behavior of the QQ plots (Figures 1a-f) in TUK1 we selected 6 tests to represent each of the 3 categories above: CcRavat (Pearson) as the collapsing test, both a simple threshold test similar to CAST (MAF Variant Threshold (“VT”, maf < 0.05) and Madsen Browning (“MB”, with polyphen weights) as the carrier tests, AMELIA as a kernel-based multivariate test, and finally two multivariate tests from Han and Pan: Sum of Squares (which is analogous to classical multivariate regression) and the adaptive Sum test, which explicitly accounts for alleles being potentially in opposite directions, but which also has a collapsing component. Our study design included methods which allowed allelic effects in opposite directions because we were studying normal subjects.

**References for Supporting Information**

1. Andrew T, Hart DJ, Snieder H, de Lange M, Spector TD, et al. (2001) Are twins and singletons comparable? A study of disease-related and lifestyle characteristics in adult women. TwinRes 4: 464-477.

2. Teslovich TM, Musunuru K, Smith AV, Edmondson AC, Stylianou IM, et al. (2010) Biological, clinical and population relevance of 95 loci for blood lipids. Nature 466: 707-713.

3. Richards JB, Rivadeneira F, Inouye M, Pastinen TM, Soranzo N, et al. (2008) Bone mineral density, osteoporosis, and osteoporotic fractures: a genome-wide association study. Lancet 371: 1505-1512.

4. Rivadeneira F, Styrkarsdottir U, Estrada K, Halldorsson BV, Hsu YH, et al. (2009) Twenty bone-mineral-density loci identified by large-scale meta-analysis of genome-wide association studies. NatGenet 41: 1199-1206.
